# Thanks to all those who reviewed for *Pilot and Feasibility Studies* in 2015

**DOI:** 10.1186/s40814-016-0050-6

**Published:** 2016-02-16

**Authors:** Gillian A. Lancaster

**Affiliations:** Postgraduate Statistics Centre, Department of Mathematics and Statistics, Lancaster University, Lancaster, UK

## Abstract

A peer-reviewed journal would not survive without the generous time and insightful comments of the reviewers, whose efforts often go unrecognized. Although final decisions are always editorial, they are greatly facilitated by the deeper technical knowledge, scientific insights, understanding of social consequences, and passion that reviewers bring to our deliberations. For these reasons, the Editor-in-Chief and staff of *Pilot and Feasibility Studies* warmly thank the 115 reviewers whose comments helped to shape the journal, for their invaluable assistance with the review of manuscripts Volume 1 (2015).

**Jonathan Adachi**

Canada

**Annabel Allison**

Switzerland

**Seyi Ladele Amosun**

South Africa

**Tom Armstrong**

USA

**John Bach**

USA

**Hee-Joon Bae**

Korea, South

**H Bickley**

UK

**Christine Blue**

USA

**Irene Bos-Touwen**

Netherlands

**David Bowrey**

UK

**Stephen Bremner**

UK

**Georges Brousse**

France

**Karen Burnell**

UK

**Michael Campbell**

UK

**Emanuele Cauda**

USA

**Philip Chilibeck**

Canada

**Jurgen Claassen**

Netherlands

**Jonathan Alistair Cook**

UK

**Peter Craig**

UK

**Gail Crimmins**

Australia

**Peter Crome**

UK

**Elizabeth Cross**

UK

**Paul Cunningham**

UK

**Brittany Dennis**

Canada

**Munyaradzi Dimairo**

UK

**Eve Madeleine Duncan**

South Africa

**Serdar Dursun**

Canada

**Richard Epstein**

USA

**Bridie Evans**

UK

**Forough Farrokhyar**

Canada

**David Fedele**

USA

**Shona Fielding**

UK

**Ivan D Florez**

Canada

**Mary Fristad**

USA

**Fadia Gamieldien**

South Africa

**Lucy Gell**

UK

**Jeremy Goldberg**

Canada

**Christine Graf**

Germany

**Colin Greaves**

UK

**Emily Haas**

USA

**Jocelyn Harris**

Canada

**Carina Heeke**

Germany

**Anne-Marie Hill**

Australia

**KY Huang**

USA

**Carmel Hughes**

UK

**Ruth Hunter**

UK

**Linda Irvine**

UK

**Zeynep Isgor**

USA

**Erica James**

Australia

**Marvin Jansen**

South Africa

**Kiran K**

India

**Sebastiana Kalula**

South Africa

**Ken Kato**

Japan

**Nikki Keeton**

South Africa

**Taeyeon Kim**

Korea, South

**Mark Kingston**

UK

**Karen Kopciuk**

Canada

**Ellen Lee**

UK

**Keir E Lewis**

UK

**Jenny Lloyd**

UK

**Chin Maguire**

UK

**Angela Masoe**

Australia

**Sunita Mathur**

Canada

**Lawrence Mbuagbaw**

Cameroon

**Hendrika Meischke**

USA

**Dan Merenstein**

USA

**Arthur Miller**

USA

**Fiona Mitchell**

UK

**L Moradveisi**

Netherlands

**Sarah Morgan-Trimmer**

UK

**Gail Anne Mountain**

UK

**Amanda Newton**

Canada

**Motoo Nomura**

Japan

**Daniel O'Connor**

Australia

**Miranda Pallan**

UK

**Gregory Parkin-Smith**

Australia

**Mershen Pillay**

South Africa

**Toby Prevost**

UK

**Cath Quinn**

UK

**Rhonda Rosychuk**

Canada

**Michael Rotondi**

Canada

**Cristin Ryan**

Ireland

**Gary Sacks**

Australia

**Zainab Samaan**

Canada

**Constantine Samaan**

Canada

**Pilar Ma Samper Ots**

Spain

**Roberto Sassi**

Canada

**Rene Schwendimann**

Switzerland

**Simon Sebire**

UK

**Krupa Shah**

USA

**Barbara Shay**

Canada

**Julius Sim**

UK

**Priya Singh**

South Africa

**Esther van Sluijs**

UK

**Kirsty Sprange**

UK

**Tracy Stecker**

USA

**Michael Stickland**

Canada

**Terri Ann Tabak**

Canada

**Natalie Taylor**

Australia

**Tobias Teismann**

Germany

**Ben Thompson**

Canada

**Mark Tully**

UK

**Katrina Turner**

UK

**Tom van Daele**

Belgium

**Thuva Vanniyasingam**

Canada

**Bart Vinck**

South Africa

**Tommy Visscher**

Netherlands

**M Vitacca**

Italy

**Theodore Wagener**

USA

**Freda Walters**

South Africa

**Sally Weinstein**

USA

**Mary White**

UK

**David White**

UK

**Francesca Zantomio**

Italy

